# Development of a personal recovery questionnaire for older adults with bipolar: a qualitative integrated knowledge translation approach

**DOI:** 10.1136/bmjopen-2024-094141

**Published:** 2026-01-19

**Authors:** Jennifer Matthewson, Elizabeth Tyler, Gillian Haddock, Steven H Jones

**Affiliations:** 1 School of Health Sciences, The University of Manchester, Manchester, UK; 2 Greater Manchester Mental Health NHS Foundation Trust, Manchester, UK; 3 Spectrum Centre for Mental Health Research, Division of Health Research, Faculty of Health and Medicine, Lancaster University, Lancaster, UK

**Keywords:** MENTAL HEALTH, Patient Participation, Aged, Old age psychiatry

## Abstract

**Abstract:**

**Objectives:**

To report on the development and refinement of a questionnaire of personal recovery for use by older adults with bipolar disorder.

**Design:**

An integrated knowledge translation approach was used to structure collaboration of individuals with clinical, research and service users. Focus groups, online meetings and online feedback were used to support information sharing.

**Participants:**

Knowledge users from across the UK including older adults with experience of bipolar, clinicians and academics.

**Primary outcome measure:**

A final draft of the Bipolar Recovery Questionnaire for Older Adults with bipolar (BRQ-OA).

**Results:**

Five service users and 15 stakeholders engaged with the study. The views and recommendations of the groups were integrated into the development of the BRQ-OA across four phases. Service users identified factors of personal recovery they felt had changed with ageing, including the impact of physical health and the importance of finding a purpose following changes to role. Collaboration with key stakeholders allowed for the development of a personal recovery questionnaire relevant to the experiences of older adults.

**Conclusions:**

An integrated knowledge translation approach successfully structured engagement with key stakeholders to allow for active and meaningful engagement. Collaboration of individuals with experience of bipolar, clinicians and academics allowed for the development of the first questionnaire of personal recovery specifically adapted for older adults with bipolar. Future research is needed to validate the BRQ-OA in older adult samples so that it can be used in mental health services and intervention studies.

**STRENGTHS AND LIMITATIONS OF THIS STUDY**

- Collaboration with service users at every stage of the research project.
- Use of focus groups allowed for collaborative discussion and open reflections with key stakeholders during the development of the questionnaire.
- The study used an integrated knowledge translation approach which allowed for structured involvement of stakeholders ensuring collaboration was meaningful and findings could be translated to practice.
- The low level of diversity across service user and research groups in terms of the gender identity and ethnicity of participants.
- The questionnaire was developed in collaboration with key stakeholders in the UK; relevance to other countries is therefore unknown.

## Introduction

 Research evidence increasingly supports the importance of a recovery-focused approach in mental health services and within therapeutic interventions.^1^ Traditional approaches focused primarily on clinical recovery are not always in line with preferences of service users and can encourage dependence on services.^2 3^ Instead, the service user-led recovery movement has called for the reframing of mental health distress away from a focus on psychiatric symptoms, towards mental health difficulties being considered as a complex phenomenon influenced by social, emotional and personal factors.^4^ The movement promoted a focus on personal recovery which emphasises the importance of connectedness, hope and optimism, identity, meaning and purpose, and empowerment.^5^ Personal recovery has further been defined as living a satisfying and contributing life alongside limitations caused by mental health experiences.^6^ Taking a recovery approach within services can enable increased hope and empowerment of individuals experiencing severe mental health difficulties.^7^ As a result, mental health policy globally has begun to emphasise the importance of services adopting a recovery-focused approach.^8^ Despite this, implementation of personal recovery approaches in mental health organisations is slow and often limited.^9^ Further work is therefore needed to increase adoption of recovery-focused strategies within services and translate research evidence to practice.

Bipolar disorder is a severe mental health diagnosis consisting of episodes of mania and/or hypomania and depression which significantly impact the functioning of an individual.^10^ Approximately 2.4% of the population will experience bipolar disorder across their lifetime with symptoms likely to persist for many years through to later life.^11 12^ Focusing on the specific needs of older adults is essential given identified differences in bipolar experiences to younger adults in terms of frequency of episodes and change to symptom experiences due to contribution of physical health and psychosocial difficulties.^13^ Older adults have been defined as people aged 60+, a population who are more likely to experience reduced functioning, increased levels of cognitive impairment and higher levels of medical comorbidity (Chet *et al*, 2017).^14^ However, research in the area of older adults with bipolar is limited and insufficient evidence is available on how to best support this group.^15^

Preliminary research has investigated how older adults with bipolar may be better supported through a recovery-focused approach.^16^ A pilot randomised control trial identified that recovery-focused therapy is a feasible and acceptable intervention which can positively impact functioning and experiences of mood symptoms in this population.^17^ A limitation of the study was the use of a personal recovery questionnaire (the Bipolar Recovery Questionnaire, BRQ) which has not been validated in an older adult population and there was no available questionnaire of personal recovery experiences of older adults with bipolar. The BRQ is a reliable and valid questionnaire developed for use with younger adults.^18^ It has successfully been used within several intervention trials for older adults with bipolar to indicate change in personal recovery experiences following support.^19 20^ However, the use of questionnaires developed and validated in working age populations may not accurately capture change in older adult populations given research evidence of differences to personal recovery with ageing.^21^ There was therefore a need to adapt the BRQ to develop a new measure which accurately captures the personal recovery experiences of older adults with bipolar.

To ensure research outcomes are relevant to service users and usable by services, co-development as part of research approach is essential.^22^ The gap between research findings and translation to use within services is commonly documented and there are concerns that research often does not reflect the needs of mental health service users.^23^ National guidance and governing bodies are increasingly advocating for researchers and services to actively collaborate with key stakeholders to increase societal impact of findings.^22^ However, few services are actively involving people with lived experiences in research and there are concerns regarding which approaches enable effective co-production.^24 25^ Integrated knowledge translation (IKT) principles have been developed to support researchers to meaningfully include the views of knowledge users.^23 26^ Knowledge users are service users, families, clinicians and policy makers where research findings have practical relevance for their work or decision making.^27^ The approach highlights that every stage in the research process is improved through collaboration with key stakeholders as it allows for development of resources specifically designed for groups who will be using them.^25^ IKT is therefore a useful approach in working collaboratively with knowledge users and improving practical relevance of findings.

The current paper reports on the adaptation of a questionnaire of personal recovery for working age adults to develop a new questionnaire suitable for use by older adults with bipolar. The paper discusses collaboration between researchers, service users, clinicians and academics to adapt and refine a questionnaire which can be used within clinical practice and future research studies. Use of an IKT approach will be discussed, including phases involved in developing and refining the questionnaire. It is hoped that use of an IKT approach will support effective research translation to use in services.

## Methods

### Study design

**Figure 1:**
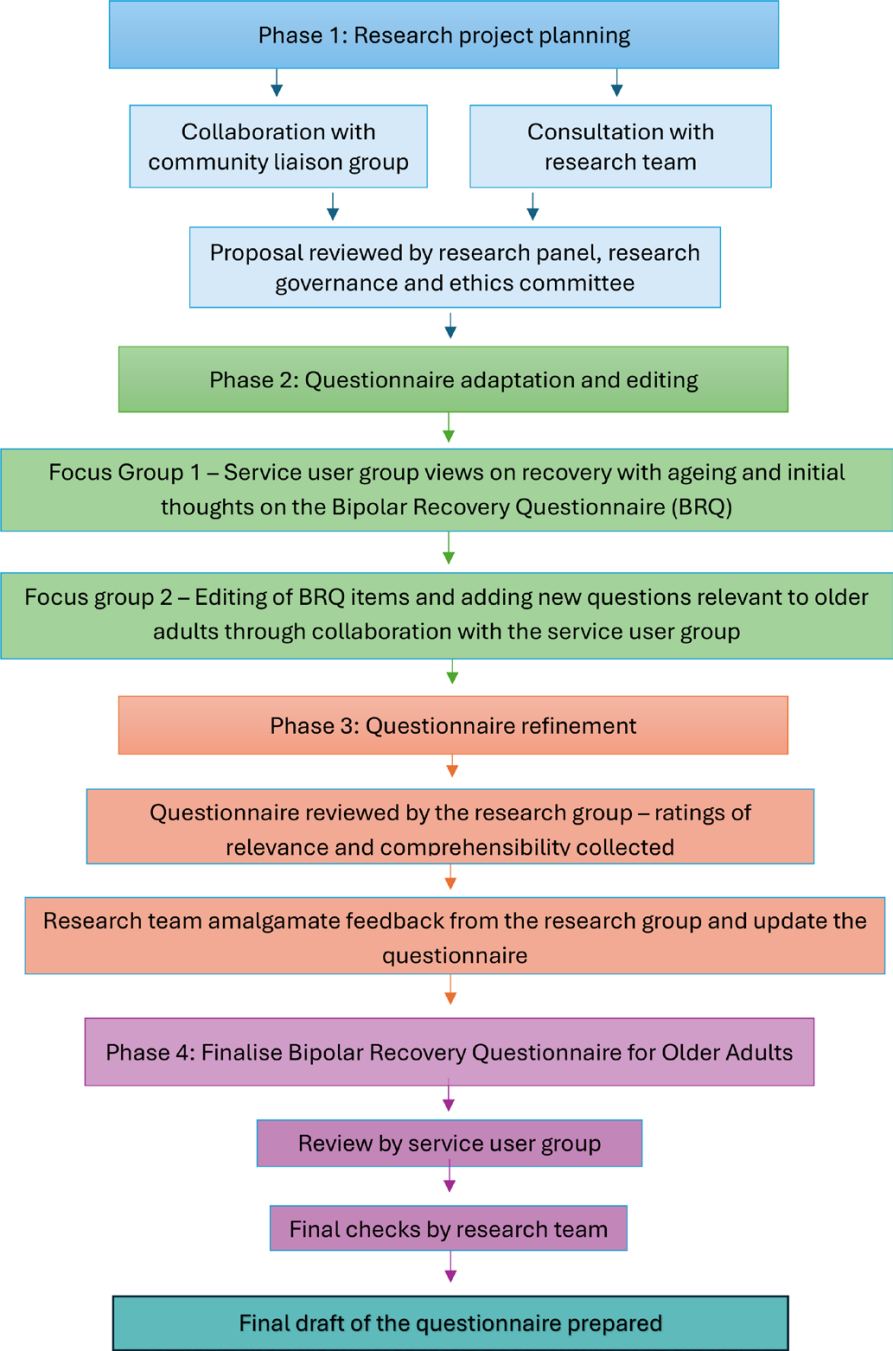
Flow chart of phases in the integrated knowledge framework to develop the Bipolar Recovery Questionnaire for Older Adults.

An IKT method was used to co-develop a questionnaire of personal recovery suitable for older adults with bipolar. Phases included use of focus groups, virtual meetings and online feedback (see figure 1). Phases were completed from June 2023 to November 2024. Focus groups were audio recorded and transcribed so key findings and recommendations could accurately be implemented. Extensive notes were taken during research meetings and online feedback from service users, clinicians and academics was recorded and collated. The research project was registered with the Open Sciences Framework (https://osf.io/cz82q/).

### Participants

Three groups were developed to participate in developing the BRQ for Older Adults (BRQ-OA):

The research team consisted of four researchers (JM, SHJ, GH and ET) and was set up prior to initiation of research process. Members of the team had experience of working clinically with older adults with bipolar and/or questionnaire development. The team developed the research question and had oversight of phases. A member of the University of Manchester community liaison group (CLG) was further enlisted to support with initial design of the study. The CLG is a group developed within the University of Manchester and consists of individuals who have experience of accessing mental health services and who work alongside researchers to advise on the development of research projects.

The service user group included four individuals who had experience of bipolar and were aged over 60 years old. Members all had previously accessed support from NHS and/or private mental health services. Individuals in the group were identified through social media and contact with NHS services in Northern England. The service user group was remunerated for their time and informed they could withdraw from the group at any stage.

The research group consisted of 15 clinicians and academics who were specialists in older adult mental health and/or supporting people with experiences of bipolar. Members of the group were identified through social media, contact with NHS services in the North of England and professional networks known to the research team.

### Measures

The original BRQ is a 36-item questionnaire of personal recovery experiences for individuals who have a diagnosis of bipolar (see online supplemental figure 1).^18^ The questionnaire has been validated in a sample aged 19–63 and found to have good test–retest and internal reliability.^18^ The measure captures personal recovery experiences including mood experiences, resources to manage mental health, access to meaningful activity and recovery as a lifelong process. The BRQ was used as a basis for development of the BRQ-OA.

Demographic information was collected from members of the service user group including age, gender, ethnicity and years since bipolar diagnosis.

### Procedures

Phase 1: Developing the research project. The research team met monthly to develop research questions, aims and procedures. Notes were taken from all meetings to track decision making and processes. Two 1-hour meetings were held with a member of the University of Manchester CLG. The individual had extensive experience as a research advisor and personal experience as a mental health service user.

A proposal was then checked by a research subcommittee at the University of Manchester consisting of senior academics. The committee provided feedback on proposed approaches, including recruitment strategies, collaboration with stakeholders and data analysis plans. Study documents were further reviewed by the University of Manchester research governance team.

Phase 2: Questionnaire development. Two focus groups, each lasting 2 hours, were held with the service user group in June 2023. Groups were facilitated by two of the research team (JM and ET) and both were attended by all service users. The aim of the focus groups was to explore the experiences of bipolar disorder of service users, their views on personal recovery in later life and to receive feedback on the original version of the BRQ.

The lead researcher (JM) called each participant individually prior to the group to identify whether they met inclusion criteria. Inclusion criteria included that all members of the focus group should be aged 60 years or over, have a diagnosis of bipolar disorder and be sufficiently able to engage with a focus group in English, due to limited funding for interpreters. An exclusion criterion was that a person currently at risk of harm or too unwell to engage would not be accepted at that time. During this call, potential group members were given information on the rationale for the project, what stages were involved and what they would be asked to do. Participants were further given time to ask questions. Each individual from the focus group was then given 24 hours minimum to consider whether they would like to take part before being asked for verbal consent in a call the following day. Verbal consent was recorded via voice recording and saved on the University of Manchester secure storage system.

During the first focus group, definitions of personal recovery were shared, including discussion of how this related to experiences of bipolar across the lifespan. The BRQ was then introduced to the group and participants were asked to reflect on overall style and individual items. Content analysis was used to identify themes from these discussions on personal recovery and ageing (see online supplemental table S1).^28^ Key topics that re-occurred were recorded and frequency of occurrence noted by author JM and outcomes reviewed by all authors. Codes were then used to draw conclusions on key topics relevant to OA experiences of personal recovery and ageing with bipolar. The outcomes of the analysis were then explored in the subsequent focus group, whereby the service user group commented on key topics and explored these in relation to the BRQ items.

During the second focus group, all items on the BRQ were reviewed in terms of readability and relevance to recovery for people aged 60 years and over. No items were removed at this stage, however, items were re-worded based on the views of service users and new items were developed by service users to cover topics relevant to older adults which were not included within the original BRQ. Relevant and adapted items from the BRQ and questions developed by the group were collated to establish a first draft of the BRQ for older adults (BRQ-OA).

Phase 3: Refining the questionnaire. The first draft of the BRQ-OA was shared with the service user and research groups via email. Members of the groups were asked to rate each of the BRQ-OA items on a scale of 1 (not at all) to 5 (a great deal) for comprehensibility and relevance to recovery in older adults. The groups were further asked for views on the questionnaire and recommendations to improve items and the questionnaire overall. Responses were shared via email and on two occasions, further qualitative feedback was shared within online meetings.

The research team met twice to discuss feedback from service user and research groups. Once feedback was collected, items with an average score of less than four for relevance to older adults were omitted from the questionnaire. Items which were scored on average as less than four for comprehensibility were re-worded using qualitative feedback on those items. Qualitative feedback on the questionnaire overall was considered and adaptations made. This resulted in a second draft of the BRQ-OA.

Phase 4: The second BRQ-OA draft was shared with the service user group for further feedback. Any changes from the previous stage were discussed in detail to ensure the measure continued to align with perspectives of the service user group and that decision-making felt appropriate. Items where wording was amended were highlighted to identify whether the service user group felt any additional amendments were needed. A final draft of the BRQ-OA was thus developed (see online supplemental figure 2).^29^

### Analysis

Qualitative feedback from focus groups and comments on initial drafts of the BRQ-OA were reviewed and key themes highlighted (see online supplemental table S1).

## Results

### Participants

The service user group (n=4) primarily identified as female (3, 75%) and were all White British. Participants ranged in age from 61 to 79. All participants had a diagnosis of bipolar disorder and most had experienced over 20 episodes of depression, mania and/or hypomania (3, 75%). Five individuals volunteered to take part within time frames, all met inclusion criteria and gave verbal consent. One individual withdrew prior to focus groups for personal reasons.

The research group (n=13) included four academics (30.77%), six clinicians (46.2%), one individual who had a dual research and clinical role (7.69%) and two individuals with experience of living with bipolar (15.39%). The group was primarily female (11, 84.62%).

### Questionnaire feedback and development

#### Phase 1

Collaboration with a CLG member during phase 1 of the project allowed for development of the research protocol (see table 1). The CLG member commented on all aspects of the research design, including recommendations for recruitment and procedures. Recommendations from the CLG were discussed by the research team and incorporated into the research protocol.

**Table 1:** Summary of discussions with a CLG member when developing the research design during phase 1

Factors discussed	Recommendations
Recruitment	Recommendations of local services who could support. Adverts to be clear that participants will be offered a safe space to engage with the project and share their views. Provision of a phone number so that individuals can speak to the researcher about any queries or concerns.
Reimbursement/remuneration	Ensuring that no participant is disadvantaged by costs of taking part. Offering appropriate remuneration for time.
Inclusivity	Broad recruitment strategies including advertising in areas across the UK. Reaching out to different communities with culturally considerate approaches. Information is to be offered in a range of formats to ensure it is accessible to different groups and the needs of individuals.
Dissemination	Sharing information in a range of formats and offering a phone call to each participant in case they prefer to receive information verbally. Sharing findings in settings recruited from so that people are more likely to see them. Use of accessible language.
Risk	Refinement of risk protocol to ensure safety of every participant. Consideration of cultural adaptations for different groups. Use of information design by CLG members which gives quick access to helpful resources.

CLG, community liaison group.

#### Phase 2

Views on personal recovery in later life were reviewed by the service user group during phase 2 of the project (see online supplemental table S1). Service users commented on continued experiences of stigma and fear of episodes, but for many, frequency and severity of mood experiences changed with ageing.

I struggled more in the last 5 years than previously

The group felt that due to having a multitude of experiences across their lifetime, they had begun to recognise patterns and effective coping strategies such as engagement with hobbies and activities which gave meaning. It was hoped that the BRQ-OA would acknowledge the extensive experiences and knowledge older adults gain as they age and how this was relevant to their recovery.

Probably got more ability to do that as you get older… you’ve got the perspective of the whole of your life

It was also felt that the questionnaire should capture the struggle that older adults have faced accessing age-suitable activities and services which may have been further impacted by age-related physical health difficulties. Participants felt that activities were in short supply and even where they were available, they were difficult to access as an older adult.

When reviewing BRQ items, service users adapted wording on seven items and recommended the addition of seven new items (see online supplemental table S2). The group commented on the need to improve clarity in some of the BRQ questions. It was felt that some of the questions could be too long and wording not easily readable. Some items were also edited to better capture factors important to personal recovery in later life, including the need to highlight recovery as an ongoing journey and how years of experience can support this.

It is nice to see life as a journey, not as fixed points that you’ve got a diagnosis, and that’s the end.

The service user group also highlighted the importance of choice in accessing resources and services, as for some, it was felt control over decision making reduced with age.

I just don’t like to involve others.

Items added to the BRQ-OA focused on factors relevant to recovery in later life such as the impact of physical health difficulties and the ability to trust others following negative experiences earlier in life. Personal factors such as perfectionism, compassion and the importance of learning from past experiences were further felt to be highly relevant.

You gain more knowledge ‘cause you’ve got more experience of blips.

The research team reviewed transcripts to confirm final edits and developed a first draft of the BRQ-OA.

#### Phase 3

When asked for feedback on the BRQ-OA, the research group commented on the importance of making the questionnaire accessible in terms of reducing number of items and adapting vocabulary used. Ratings of relevance were used to remove four items which had an average Likert scale rating of less than 4. Topics of questions removed included engagement with challenging tasks, management of mood fluctuations and the impact of experiences on trust. Four items were also re-worded due to having an average comprehensibility score of less than 4. Recommended edits to improve comprehensibility included simplification of wording, making statements more general to be more applicable to a range of individuals and being clearer on the factor of focus within the question. Feedback from the research group and service users was discussed by the research team and edits made across two 1-hour meetings. This led to the development of the 39-item BRQ-OA.

#### Phase 4

During phase 4, the research team shared the final version of the BRQ-OA which was approved by members of the service user group. Due to larger numbers of researchers involved, it was felt important that the service user group had final say on all changes made. Service users gave two suggestions on editing to wording which were incorporated into the final BRQ-OA. The BRQ-OA then underwent a process of psychometric validation.^29^

## Discussion

The current paper reports on the development of a personal recovery questionnaire for older adults with bipolar, the BRQ-OA. Use of an IKT approach allowed for collaboration with key stakeholders at each stage of the research process. Across four phases, the research team worked with the service user and research groups to gain feedback and insight on how to best develop and refine the BRQ-OA. Service users reflected on key changes in their views of personal recovery with ageing and how this could be captured by the BRQ-OA. Main themes included the impact of physical health difficulties, the importance of choice and the influence of experience gained across the lifespan. Four items were omitted and four items re-worded based on feedback from the research group and overall feedback on the questionnaire was used to increase quality and relevance of the BRQ-OA. During a final review of the questionnaire, knowledge users felt that their recommendations had been accurately followed, resulting in questions on the BRQ-OA being relevant to their age group, experiences of bipolar and views of personal recovery.

National guidance indicates that all mental health service users have the right to be directly involved with research and development of resources; however, application of co-production is limited in practice.^30^ The current paper used an IKT approach to collaboratively involve stakeholders in processes and develop a questionnaire which can support use of recovery-focused approaches in services and research on the development of recovery-focused interventions for older adults with bipolar. While alternative collaborative approaches are available, IKT was viewed as most appropriate given its focus on development of knowledge for practical application.^31^ The IKT approach allowed for structured involvement of knowledge users at each stage. Previous research has used an IKT approach to develop interventions, reporting guidelines and health promotion initiatives.^32 33^ To our knowledge, this is the first paper to use an IKT approach in the development of a questionnaire.

The BRQ-OA is a 39-item questionnaire of personal recovery experiences adapted from the BRQ for use with older adults who have bipolar. In line with knowledge user guidance, the questionnaire captures key areas of recovery including knowledge gained through a lifetime of experiences, the importance of choice and the need for meaningful activity in later life. The BRQ-OA can be used within services at the start of therapeutic interventions to encourage conversations between service users and clinicians on their unique views on personal recovery and how services can support them to reach meaningful goals. Further completion of the questionnaire across intervention can indicate progress, highlight whether adaptations to approaches are needed and can be recorded as an outcome questionnaire. Similarly, the BRQ-OA can be adopted in future research to support development of interventions specifically designed for older adults.^17^ Before the BRQ-OA can be adopted by services, future research can be completed to ensure the BRQ-OA is accurately capturing concepts commonly related to recovery. An IKT approach allows for the development of a tool which aligns with the views of key stakeholders. Further comparison of the measure with validated tools which capture concepts related to recovery can then indicate face and content validity of the questionnaire in capturing personal recovery experiences. Responses on the BRQ-OA will be compared with measures of mental health and well-being commonly associated with recovery in the literature. This can then support the paper in being both relevant to service user experiences and accurate in capturing concepts relevant to recovery. Once further validated, the BRQ-OA can be implemented as a collaboratively produced questionnaire of personal recovery.^29^

Knowledge users commented on aspects of mental health and personal recovery that changed with ageing. Research supports the unique experience of bipolar in older adults compared to working age adults, including the impact of medical comorbidities and changes in experiences of mood episodes.^16 34^ Service users reflected on the growing importance of hobbies with age as other meaningful activities, such as employment, decrease. Other research has similarly highlighted the impact of change in social role with age and the ongoing need for purpose.^16^ Identity and the importance of choice and independence were further discussed by knowledge users, highlighting to services the importance of considering how older adults may feel their independence and identity have altered.^21^ Other themes identified in the current study, including the role of perfectionism, are less commonly discussed in older adult research; however, they have been highlighted in research on bipolar disorder experiences of younger people.^35 36^ This highlights the nature of personal recovery as a unique concept which should be explored with everyone during their access to services. The BRQ-OA may be used as a helpful tool to encourage conversations and aid the development of personally relevant aims for intervention.

### Strengths and limitations

A strength of design was use of multiple focus groups allowing for discussion and collaborative reflections as recommended in IKT design. A limitation is that the research group submitted recommendations and feedback on BRQ-OA items independently. On some occasions, feedback was contradictory, making it challenging for the research team to accurately represent the views of each contributor. Contacting the research group via email enabled a range of stakeholders to share their views; however, the opportunity for discussion may have allowed for consensus on editing the questionnaire and aided refinement. The research team managed contradictory feedback through discussion of potential impact, reference to the evidence base and by discussing all changes with the service user group.

A highlight of the current study is the involvement of knowledge users at each stage in the research process; however, the imbalance of numbers across the service user (6) and researcher groups (15) should be noted. The team aimed to collect detailed qualitative information from service users and all changes to the BRQ were developed by the service user group to ensure the BRQ-OA aligned with the views of individuals who have experience of living with bipolar disorder and accessing services. The research group then gave valuable ideas on how changes may support use in clinical settings. However, all changes suggested and decisions made were reviewed with the service user group before finalising measures to ensure a collaborative approach. Use of an IKT approach therefore allowed for meaningful engagement with service users and clinicians to bridge the gap between research and translation to practice.^37^

Clinicians and academics highlighted the importance of questionnaire brevity to enable use in services. As most BRQ-OA items were classed as relevant, the final draft of the BRQ-OA has a relatively high number of items. Further research is needed to explore the psychometric properties of the scale which may allow for item reduction where appropriate to improve practical usability in clinical settings. A further limitation of the study was the lack of diversity in both the service user and research groups. Those involved in the research were primarily White British and female. Recommendations shared may therefore not be in line with the views of individuals from different cultural backgrounds, ethnicities or gender identities. Limited diversity in knowledge user groups is not unusual within the research area.^32^ Future research using an IKT approach should therefore identify alternative recruitment strategies making participation more accessible and attractive to diverse groups of individuals to ensure findings better represent knowledge users. This could include contacting charities and community leaders who support minority groups. Information shared on the study could also be adapted so it is more accessible and attractive to people from different cultural and ethnic backgrounds. The BRQ-OA was also solely developed within the UK; future iterations of the questionnaire may be needed for use in different countries and communities.

A highlight of the project; however, is focus on the needs of older adults who are often underrepresented across research.^38^ There is currently a dearth of evidence looking at the needs and priorities of older adults with bipolar disorder.^13^ This is of great concern given evidence that older adults have a lower treatment response to therapeutic interventions and questionnaires validated with younger adults may be inaccurate in capturing the experiences of people in later life.^39^ It is therefore a significant strength of the study that the BRQ-OA can be used to imitate future research in the area so that older adults with bipolar are better supported.

Though thought was given within the BRQ-OA to engagement with activities that keep participants well and feelings of choice over life, there was no direct consideration of protective factors which may support well-being across psychological intervention. Research highlights the importance of comprehensively addressing factors which build strength in service users’ lives and help them to access treatment.^40^ Further iterations of the measure may seek to consider whether increased inclusion of questions around protective factors may support use of the measure in clinical settings and understanding of recovery over time.

## Conclusions

An IKT approach was successfully used to develop a questionnaire of personal recovery for use by older adults with bipolar. The use of a collaborative approach with service users, academics and clinicians allowed for sharing of perspectives on adapting the questionnaire so it was relevant to the experiences of adults in later life and acceptable to those who will use it. Use of IKT ensured that knowledge users were actively engaged at each stage of the research project. To our knowledge, this is the first use of IKT in developing a questionnaire of personal recovery experiences. Further research is underway to validate the BRQ-OA so that it can be used in mental health services and intervention studies.^29^

## Supplementary material

10.1136/bmjopen-2024-094141 online supplemental file 1

## Data Availability

Data are available on reasonable request.
