# Supplementary material for: Development of a personal recovery questionnaire for older adults with bipolar: a qualitative integrated knowledge translation approach
Source: BMJ Open. 2026 Jan 19;16(1):e094141. doi: 10.1136/bmjopen-2024-094141 (PMC12820841; doi:10.1136/bmjopen-2024-094141)
Supplement: online supplemental file 1 [file bmjopen-16-1-s001.docx]

Supplementary Material

Table S1. Service user group views on personal recovery as older adults and on the BRQ.

| Objective | Findings | Supporting quotes |
| --- | --- | --- |
| Changes in bipolar experiences over time | Continued fear of hospitalisation  Changes in frequency and severity of symptoms with age – for some this meant a decrease in symptoms while others felt they had increased  Improved strategies to manage symptoms  Challenges of getting psychological help from later life services  Improvements in mental health knowledge vs ongoing stigma  Increase in physical health difficulties and impact on mood | “that’s kind of a fear about going, having to go back in” (PPT 3)  “I’d say they are longer apart… less frequent” (PPT 3)  “I struggled more in the last 4 years than previously” (PPT 1)  “You find different ways to manage them over time” (PPT 2)  “Probably got more ability to do that as you get older… you’ve got the perspective of the whole of your life” (PPT 2)  ”There’s virtually no one who can help you” (PPT 4)  “Psychological resources are in very short supply, they’re like gold dust” (PPT 2)  “I think society has maybe got a bit more open” (PPT 2)  “People still shy away from… talking with somebody about mental health issues” (PPT 3)  “I think there is just a lot of prejudice if you’ve got a severe mental illness”  “I’ve got diabetes, heart problems, and it doesn’t help” (PPT 1) |
| Personal recovery in later life | Importance of hobbies  The need to have meaning and purpose  Recovery as a journey  Diagnosis as part of identity  Importance of independence  Negative impact of perfectionism | “I’m fine now. I’ve got a lot of big hobbies” (PPT 1)  “My whole depression was about a loss of meaning… so it’s been a process of getting purpose back” (PPT 2)  “I’ve got to keep my mind active” (PPT 1)  “It all goes to that question of self-worth, doesn’t it?” (PPT 3)  “It’s nice to see life as a journey, not as fixed points that you’ve got a diagnosis, and that’s the end” (PPT 2)  “Being bipolar is right through me, like a stick of rock... not just a limiting label but in some ways it’s freed me” (PPT 2)  “If you’ve lived with it for a long time, it is part of you” (PPT 4)  “If you depend a lot on others… then it’s an indication that maybe you haven’t recovered quite so well because you’re not yet independent” (PPT 3)  “I like my independence” (PPT 1)  “Perfectionism, which I feel I suffer from… it does take a toll on you as a person” (PPT 4) |
| Changes in personal recovery across the lifespan | Difficulties associated with ageing  Increased learning and symptom recognition with age  Change in access to meaningful activities  Increase in physical health difficulties  Changes in understanding where experiences come from  Impact of experiences across life on decision making  Highlighting importance of experiences | “all the changes you have… you know, adjusting to that”  “circumstances are changing constantly… you don’t know what’s what” (PPT 4)  “I hope I have learnt from my past and I hope if I get down again… I will recognise it” (PPT2)  “I am more or less house bound” (PPT 1)  “As you’re older, if some activities are more youth focused… can feel a bit reluctant to go and join them” (PPT2)  “If you’re in constant pain, that’s very limiting” (PPT 4)  “With more experiences I put it (episodes) down to other reasons” (PPT 4)  “Sadly you gain more knowledge ‘cause you’ve got more experience of blips and how treatment has worked out” (PPT 2)  “Because that’s what we’ve got as older people, we’ve got the experience” (PPT 2) |
| Changes to items | Contentment with self in relation to having a bipolar diagnosis  Importance of access to mental health services and education as part of resources  Highlighting family and friends as sources of support  Re-wording to ensure clarity of topic  Importance of choice in asking for support from others | “I can still be contented.. even though I have a diagnosis or a history of, of illness” (PPT 2)  “’Cause resources might be education or other things as well as services” (PPR 2)  “Because family keep you as safe as they can” (PPT 2)  “I feel as though it needs some kind of verification on it” (PPT 3)  “I just don’t like to involve others” (PPT 1)  “having the support if you did want to involve them” (PPT 3)  “I’ve had negative reactions… you don’t always trust people enough to them anything about mental health” (PPT 2) |

Table S2. Changes made to BRQ and final BRQ-OA items

| Original BRQ item | Changes to item | Final BRQ-OA item |
| --- | --- | --- |
| 1. I struggle to make sense of the experiences I have had | Remained the same | 1. I struggle to make sense of the experiences I have had |
| 1. I have the resources to effectively manage my health | Remained the same | 1. I have the resources to effectively manage my health |
| 1. I am content with who I am as a person | Remained the same | 1. I am content with who I am as a person |
| 1. I have little control over my mood | Remained the same | 1. I have little control over my mood |
| 1. I avoid taking on challenges in life that matter to me | Rated as low for comprehensibility  Change of wording | 1. I avoid things that are difficult in life even if they matter to me |
| 1. I see recovery as a lifelong process | Remained the same | 1. I see recovery as a lifelong process |
| 1. I think differently about some of my experiences now compared with when they frist occurred | Remained the same | 1. I think differently about some of my experiences now compared with when they first occurred |
| 1. I can access the help I need in order to stay well | Change of wording within focus group | 1. I can access the services I need in order to stay well |
| 1. My experiences have made me the person I am today | Remained the same | 1. My experiences have made me the person I am today |
| 1. I recognise when I am in situations that aren’t good for my wellbeing | Remained the same | 1. I recognise when I am in situations that aren’t good for my wellbeing |
| 1. I am able to engage in a range of activities that are personally meaningful to me | Change of wording within the focus group | 1. I am able to engage in a range of activities that are personally meaningful to me at my age |
| 1. Recovery means forgetting about my mental health problems | Remained the same | 1. Recovery means forgetting about my mental health problems |
| 1. I am unsure about the reasons behind some of the experiences I have had | Rated as low for comprehensibility. Change of wording to “I am unsure why some of my experiences have happened” | 1. I am unsure why some of my experiences have happened |
| 1. I feel in control of the things that happen in my life | Edited as part of focus group but then rated as low for comprehensibility. Change of wording to original. | 1. I feel in control of the things that happen in my life |
| 1. I am productive in the things in life I engage in | Remained the same | 1. I am productive in the things in life I engage in |
| 1. I depend on others to maintain my own wellbeing | Remained the same | 1. I depend on others to maintain my own wellbeing |
| 1. I feel confident enough to get involved in the things in life that interest me | Remained the same | 1. I feel confident enough to get involved in the things in life that interest me |
| 1. I can have mood experiences and still get on with my life | Remained the same | 1. I can have mood experiences and still get on with my life |
| 1. I can see where certain experiences I have had have come from | Wording changed in focus group. | 1. As I’ve got older, I can see where certain mental health experiences have come from |
| 1. I am able to decide when I need support from others in order to maintain my wellbeing | Remained the same | 1. I am able to decide when I need support from others in order to maintain my wellbeing |
| 1. I get little personal satisfaction out of the things in life I am involved in | Remained the same | 1. I get little personal satisfaction out of the things in life I am involved in |
| 1. I have the knowledge to make informed decisions concerning treatment for my mental health | Wording changed in focus group. | 1. I have knowledge from previous experiences to make informed decisions concerning treatment for my mental health |
| 1. I am unhappy with the person I have become | Remained the same | 1. I am unhappy with the person I have become |
| 1. I sometimes let me mood fluctuate if I have important tasks to do | Removed – irrelevant |  |
| 1. The high standards I set myself are unrelated to fluctuations in my mood | Removed – irrelevant  Also rated as low for comprehensibility |  |
| 1. I play a central role in maintaining my own wellbeing | Remained the same | 1. I play a central role in maintaining my own wellbeing |
| 1. I have the ability to achieve my goals in life | Remained the same | 1. I have the ability to achieve my goals in life |
| 1. My ability to make informed choices about treatment is supported by my friends and family | Wording changed to in focus group. | 1. I have support from friends and family to make informed choices about treatment where I ask for it |
| 1. I find it hard to engage in a range of activities that are valuable to me | Remained the same | 1. I find it hard to engage in a range of activities that are valuable to me |
| 1. I can still be in recovery even if I experience mood episodes in the future | Remained the same | 1. I can still be in recovery even if I experience mood episodes in the future |
| 1. Understanding where my mood experiences come from help me manage them | Wording changed in focus group. | 1. Understanding what causes my mood experiences can help me to manage them |
| 1. I have little control over the important decisions in my life | Remained the same | 1. I have little control over the important decisions in my life |
| 1. I am able to engage in a range of activities that are valuable to wider society | Remained the same | 1. I am able to engage in a range of activities that are valuable to wider society |
| 1. The knowledge I have gained enables me to look after myself | Wording changed in focus group. | 1. My experiences and the knowledge I have gained over time enable me to look after myself now |
| 1. The activities I do make a difference to others | Remained the same | 1. The activities I do make a difference to others |
| 1. Being in recovery means that everything has to be going well in every aspect of my life | Remained the same | 1. Being in recovery means that everything has to be going well in every aspect of my life |
| Questions added within OA focus group |  |  |
| 1. Over the years I have learnt to be compassionate with myself | Remained the same | 1. Over the years I have learnt to be compassionate with myself |
| 1. I am unhappy unless things are perfect | Remained the same | 1. I am unhappy unless things are perfect |
| 1. I am able to access support from those around me to stay well | Remained the same | 1. I am able to access support from those around me to stay well |
| 1. I have learnt from my past | Remained the same | 1. I have learnt from my past |
| 1. My physical health stops me from being able to do things that are important to me | Remained the same | 1. My physical health stops me from being able to do things that are important to me |
| 1. I struggle to trust others due to my experiences over time | Removed – irrelevant |  |
| 1. I feel able to engage with my community despite the changes in my role as I have grown older | Removed - irrelevant |  |

**Supplementary Figure 1: Bipolar Recovery Questionnaire**
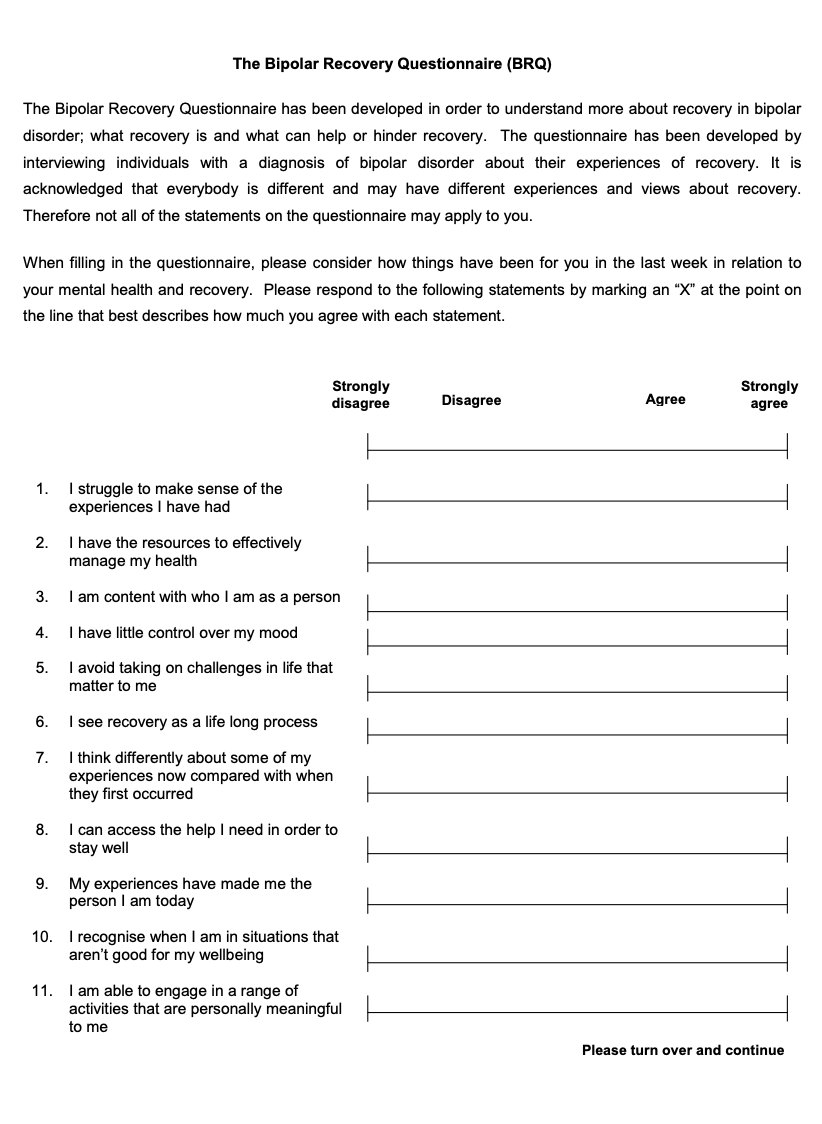


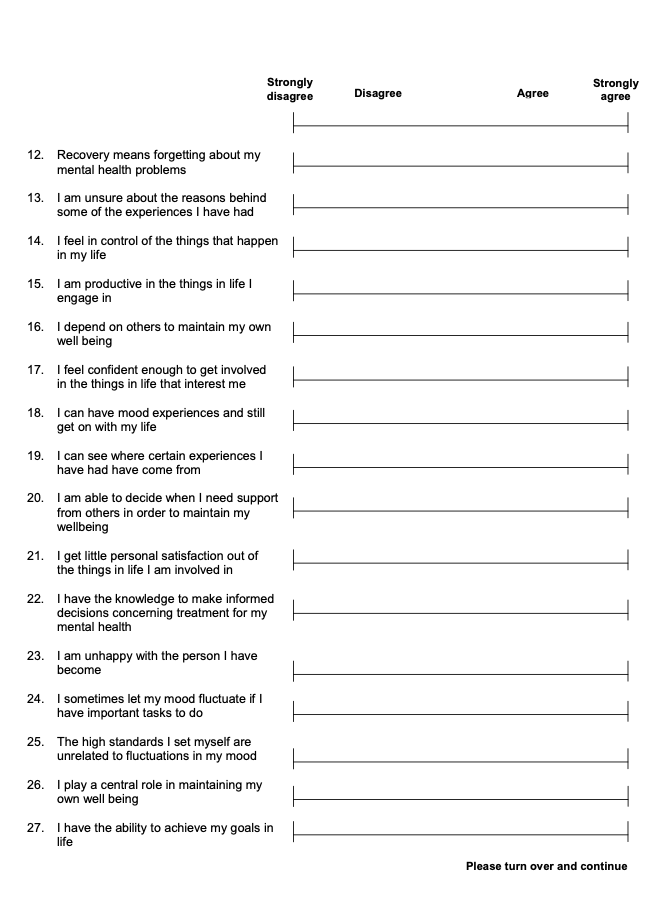


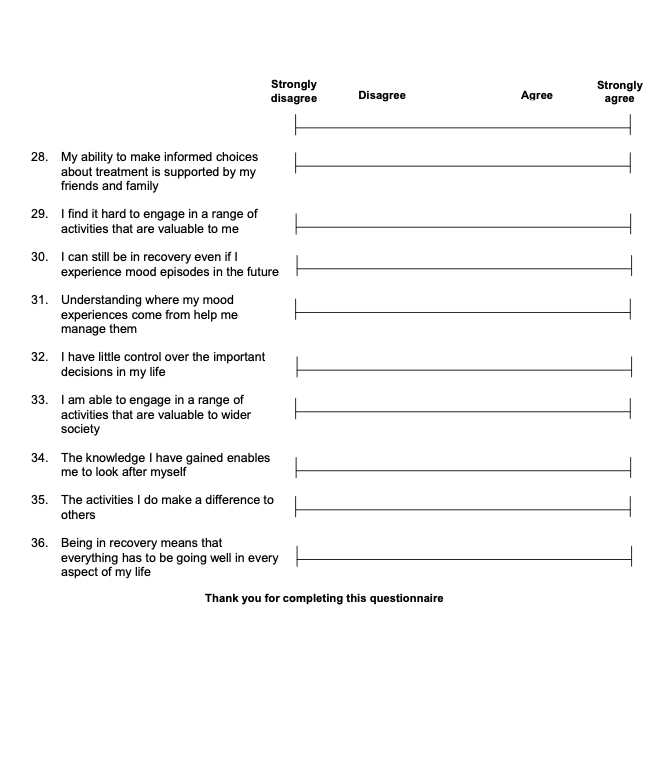


**Supplementary figure 2 – Bipolar Recovery Questionnaire for Older Adults**

**The Bipolar Recovery Questionnaire for older adults (BRQ-OA)**

The Bipolar Recovery Questionnaire for older adults has been developed in order to understand more about recovery in bipolar disorder; what recovery is and what can help or hinder recovery. The questionnaire has been developed by interviewing individuals with a diagnosis of bipolar disorder about their experiences of recovery. It is acknowledged that everybody is different and may have different experiences and views about recovery. Therefore, not all of the statements on the questionnaire may apply to you.


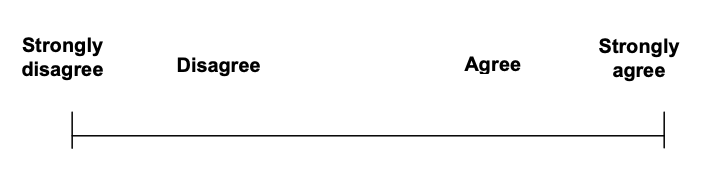
When filling in the questionnaire, please consider how things have been for you in the last week in relation to your mental health and recovery. Please respond to the following statements by marking an “X” at the point on the line that best describes how much you agree with each statement.

1. I struggle to make sense of the

experiences I have had

1. I have the resources to effectively

manage my health

1. I am content with who I am as

a person

1. I have little control over my mood

1. I avoid things that are difficult in

life even if they matter to me

1. I see recovery as a life long process
2. I think differently about some of my experiences now compared with when they first occurred
3. I can access the services I need in order to stay well
4. My experiences have made me the person I am today


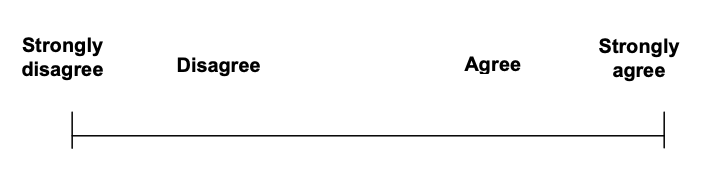


1. I recognise when I am in situations that aren’t good for my wellbeing
2. I am able to engage in a range of activities that are personally meaningful to me at my age
3. Recovery means forgetting about my mental health problems
4. I am unsure why some of my experiences have happened
5. I feel in control of the things that happen in my life
6. I am productive in the things in life I engage in
7. I depend on others to maintain my own well being
8. I feel confident enough to get involved in the things in life that interest me
9. I can have mood experiences and still get on with my life
10. As I’ve got older, I can see where certain mental health experiences have come from

**Please turn over and continue**


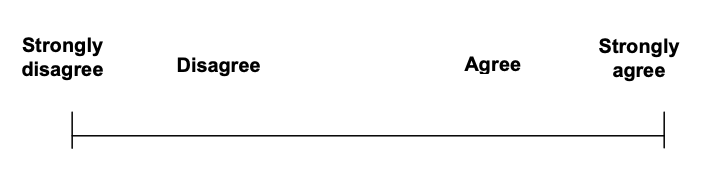


1. I am able to decide when I need support from others in order to maintain my wellbeing
2. I get little personal satisfaction out of the things in life I am involved in
3. I have knowledge from previous experiences to make informed decisions concerning treatment for my mental health
4. I am unhappy with the person I have become
5. I play a central role in maintaining my own well being
6. I have the ability to achieve my goals in life
7. I have support from friends and family to make informed choices about treatment where I ask for it
8. I find it hard to engage in a range of activities that are valuable to me
9. I can still be in recovery even if I experience mood episodes in the future

**Please turn over and continue**


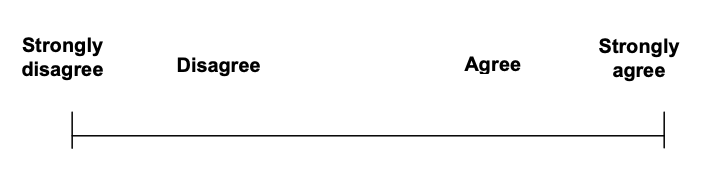


1. Understanding what causes my mood experiences can help me to manage them
2. I have little control over the important decisions in my life
3. I am able to engage in a range of activities that are valuable to wider society
4. My experiences and the knowledge I have gained over time enable me to look after myself now
5. The activities I do make a difference to others
6. Being in recovery means that everything has to be going well in every aspect of my life
7. Over the years I have learnt to be compassionate with myself
8. I am unhappy unless things are perfect
9. I am able to access support from those around me to stay well
10. I have learnt from my past

**Please turn over and continue**


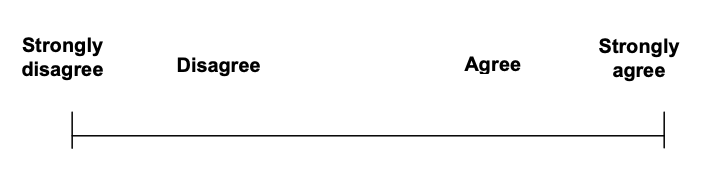


1. My physical health stops me from being able to do things that are important to me

**Thank you for completing this questionnaire**
