# Supplementary material for: Development of a personal recovery questionnaire for older adults with bipolar: a qualitative integrated knowledge translation approach
Source: BMJ Open. 2026 Jan 19;16(1):e094141. doi: 10.1136/bmjopen-2024-094141 (PMC12820841; doi:10.1136/bmjopen-2024-094141)
Supplement: Supplementary file 2 [file bmjopen-2024-094141.reviewer_comments.pdf]

PEER REVIEW HISTORY

BMJ Open publishes all reviews undertaken for accepted manuscripts. Reviewers are asked to complete a checklist review form and are provided with free text boxes to elaborate on their assessment. These free text comments are reproduced below.

ARTICLE DETAILS

Title (Provisional)

Development of a personal recovery questionnaire for older adults with bipolar: a qualitative integrated knowledge translation approach

Authors

Matthewson, Jennifer; Tyler, Elizabeth; Haddock, Gillian; Jones, Steven H

VERSION 1 - REVIEW

**Reviewer**	**1**
**Name**	**Steardo, Luca**
**Affiliation**	**Magna Graecia University of Catanzaro**
**Date**	**07-Oct-2024**
**COI**	**None**

The scientific article presents several strengths as well as areas that could benefit from improvement. Here's a summary based on the document provided:

Strengths

Integrated Knowledge Translation (IKT) Approach: The study used an IKT approach, which involved collaboration with service users, clinicians, and academics at each stage. This inclusive method ensured that the resulting questionnaire was relevant and meaningful to older adults with bipolar disorder.

Stakeholder Involvement: The research involved a range of stakeholders, including individuals with lived experience of bipolar disorder, clinicians, and academics. This engagement added depth and real-world relevance to the study.

Focus on Underrepresented Population: The study addressed the gap in research for older adults with bipolar disorder, a group that is often underrepresented in mental health research.

Comprehensive Methods: The use of focus groups, online meetings, and feedback for developing the questionnaire demonstrated a thorough approach to capturing diverse perspectives.

Development of a New Tool: The creation of the Bipolar Recovery Questionnaire for Older Adults (BRQ-OA) is a novel contribution, providing a tool specifically tailored for assessing recovery in an older population with bipolar disorder.

Attention to Recovery: The article emphasizes personal recovery experiences, which align with modern mental health approaches focusing on patient-centered care and personal empowerment.

Areas for Improvement

Methodological Limitations:

Low Diversity of Participants: The participant pool lacked diversity in terms of gender identity and ethnicity, which limits the generalizability of the findings. Future research should seek to involve a more diverse group of knowledge users and stakeholders.

Geographic Limitation: Since the questionnaire was developed based on collaboration with UK-based stakeholders, its applicability to other cultural and healthcare settings is uncertain. Cross-cultural validation studies are needed.

Independent Feedback: The research advisory group independently submitted recommendations and feedback on BRQ-OA items. The lack of interactive discussion might have led to contradictory feedback, which could have been mitigated through group discussions to achieve consensus.

Study Limitations:

Questionnaire Length: The final draft of the BRQ-OA contains a relatively high number of items. Although most were rated as relevant, further research could explore reducing the number of items to improve their practicality in clinical settings.

Relevance to Non-UK Settings: Based on the UK population, the questionnaire's development raises questions about its relevance and applicability to older adults in different countries and cultural contexts.

Areas for Future Improvement:

Psychometric Validation: The article notes the need for future research to validate the BRQ-OA. This step is crucial for establishing the tool's reliability and validity in capturing personal recovery experiences in older adults.

Alternative Recruitment Strategies: Future research should explore more inclusive recruitment strategies to ensure participation from individuals of various cultural, ethnic backgrounds, and gender identities. Collaborations with charities and community leaders could be a strategy to improve diversity.

Overall, while the article presents a strong, collaborative approach to developing a new tool for assessing recovery in older adults with bipolar disorder, it would benefit from addressing

participant diversity, validating the tool in non-UK contexts, and considering methodological refinements

---

Reviewer **2**

Name **Tusconi, Massimo**

Affiliation **Universita degli Studi di Cagliari, Medical Science and Public Health**

Date **09-Oct-2024**

COI **None**

Dear Editor,

I appreciate the opportunity to review the manuscript entitled:

"Development of a personal recovery questionnaire for older adults with bipolar: a qualitative integrated knowledge translation approach"

I commend the authors for describing this critical and timely issue. The paper is interesting and well-written; however, I would like to highlight some issues that merit revision:

In the manuscript, it is not detectable whether an assessment was made of what might be a protective factor through psychotherapy and counseling intervention, which people often use without prescription to prevent or alleviate symptoms at the onset. I would ask the authors if this was evaluated during the survey and add a short paragraph on this issue; if data are unavailable, add them to the limitations.

Reviewer **3**

Name **Banfield, Michelle**

Affiliation **Australian National University**

Date **20-May-2025**

COI **None**

The measurement of recovery from the perspective of people with lived experience is an under-researched area. Much greater emphasis is needed on defining and properly evaluating recovery from views other than clinical and academic. This paper is an important contribution in that space, but I do have some suggestions to improve clarity.

1. In the introduction, it is not until around page 5-6 that we get some sense of the authors' definition of "older adults" and why they think recovery parameters may be different for this

group (mention of beyond working age). It would help the siting of the research to introduce this much earlier.

2. Throughout the manuscript, the language used to describe the participants who brought lived experience of bipolar disorder varies, with inconsistent descriptions of who took part where. In the methods, 4 participants are described as "knowledge users" and a distinct group from the "advisory group", but the latter is then described as including "experts by experience" with no elaboration on what that means. Knowledge users and experts by experience appear to be used interchangeably across all elements of the manuscript without clear definition of either, with the term "service user" also sometimes appearing. Further, the discussion section shifts to describing knowledge users as involved in all stages, which is not supported by the flow diagram. Suggest revising all parts to clearly define and consistent apply terms relating to lived experience.

3. The methods are fairly well described, with the exception of the analysis section. Content analysis is part of a broad family of methods that can be implemented in a number of ways - a reference to a specific process would help. The analysis needs to be further unpacked with details such as who conducted the analyses, did they involve frequencies, were they checked with members to assist with interpretation etc.

4. The main text of the results section is quite light on content and relies on cross-referencing to tables for an understanding of the key findings. This section would benefit from further incorporation of findings and perhaps some of the quotes within the narrative to give a deeper sense of the content of themes and feedback.

5. A key limitation of the study is the disproportionate lived experience versus clinical and academic involvement. The study is positioned as using IKT to integrate a range of views, but the authors do not explicitly address the unbalanced numbers and power dynamics apparent in 4 (+2?) people with bipolar disorder versus 15 advisory group members + 4 researchers from clinical and academic backgrounds. As the methods further describe the researchers conducting analyses and making decisions regarding contradictory feedback, the strength of the lived experience contributions as "collaboration" is questionable as they appear to have been filtered through clinical/academic lenses at each stage. This needs to be acknowledged throughout the manuscript and particularly as a limitation of the study.

6. Page 9, please revise the phrasing "...explore the bipolar experiences..." to, for example, "explore the experiences of bipolar disorder".

7. Page 3 Limitations please revise "expert by experiences" to "experts by experience".

8. Although subject of a separate paper under development, the comment on p13 regarding establishing validity needs some unpacking. The argument for the current work is that no suitable measure of recovery for older adults with bipolar disorder exists, so how will cross-validation and reliability be undertaken as separate to the face validity of the co-developed

measure? Requiring traditional psychometrics appears to undermine the value of the IKT process.

VERSION 1 - AUTHOR RESPONSE

Reviewer 1	
Methodological limitations	
Low Diversity of Participants: The participant pool lacked diversity in terms of gender identity and ethnicity, which limits the generalizability of the findings. Future research should seek to involve a more diverse group of knowledge users and stakeholders.	See pages 15 – 16<br><br>"A further limitation of the study was the lack of diversity in both the service user and research groups. Those involved in the research were primarily White British and female."
Geographic Limitation: Since the questionnaire was developed based on collaboration with UK-based stakeholders, its applicability to other cultural and healthcare settings is uncertain. Cross-cultural validation studies are needed.	Further comments added to limitations section (pages 15 and 16)<br><br>"The BRQ-OA was also solely developed within the UK, future iterations of the questionnaire may be needed for use in different countries and communities."
Independent Feedback: The research advisory group independently submitted recommendations and feedback on BRQ-OA items. The lack of interactive discussion might have led to contradictory feedback, which could have been mitigated through group discussions to achieve consensus.	Discussed in limitations section (page 15)<br><br>"however, the imbalance of numbers across the service user (6) and researcher groups (15) should be noted. The team aimed to collect detailed qualitative information from service users and all changes to the BRQ were developed by the service user group to ensure the BRQ-OA aligned with the views of individuals who have experience of living with bipolar disorder and accessing services. The research group then gave valuable ideas on how changes may support use in clinical settings, however, all changes suggested, and decisions made were reviewed with the service user group before finalising measures to ensure a collaborative approach."
Study Limitations	
Questionnaire Length: The final draft of the BRQ-OA contains a relatively high number of items. Although most were rated as relevant, further research could	Additional discussion in limitations (please see page 15)

explore reducing the number of items to improve their practicality in clinical settings.	"Further research is needed to explore the psychometric properties of the scale which may allow for item reduction where appropriate to improve practical usability in clinical settings."
Relevance to Non-UK Settings: Based on the UK population, the questionnaire's development raises questions about its relevance and applicability to older adults in different countries and cultural contexts.	See limitations (page 15 and 16)<br><br>"A further limitation of the study was the lack of diversity in both the service user and research groups. Those involved in the research were primarily White British and female. Recommendations shared may therefore not be in line with the views of individuals from different cultural backgrounds, ethnicities or gender identities. Limited diversity in knowledge user groups is not unusual within the research area [30]. Future research using an IKT approach should therefore identify alternative recruitment strategies making participation more accessible and attractive to diverse groups of individuals to ensure findings better represent knowledge users. This could include contacting charities and community leaders who support minority groups. Information shared on the study could also be adapted so it is more accessible and attractive to people from different cultural and ethnic backgrounds. The BRQ-OA was also solely developed within the UK, future iterations of the questionnaire may be needed for use in different countries and communities."

Psychometric Validation: The article notes the need for future research to validate the BRQ-OA. This step is crucial for establishing the tool's reliability and validity in capturing personal recovery experiences in older adults.	As discussed in limitations section and plans for future research (Page 15) "Further research is needed to explore the psychometric properties of the scale which may allow for item reduction where appropriate to improve practical usability in clinical settings."
Alternative Recruitment Strategies: Future research should explore more inclusive recruitment strategies to ensure participation from individuals of various cultural, ethnic backgrounds, and gender identities. Collaborations with charities and community leaders could be a strategy to improve diversity.	As discussed in limitations section (top of page 16)

Reviewer 2	
In the manuscript, it is not detectable whether an assessment was made of what might be a protective factor through psychotherapy and counselling intervention, which people often use without prescription to prevent or alleviate symptoms at the onset. I would ask the authors if this was evaluated during the survey and add a short paragraph on this issue; if data are unavailable, add them to the limitations.	Paragraph added to limitations, please see page 16.<br><br>"Though thought was given within the BRQ-OA to engagement with activities that keep participants well and feelings of choice over life, there was no direct consideration of protective factors which may support wellbeing across psychological intervention. Research highlights the importance of comprehensively addressing factors which build strength in service user's lives and help them to access treatment [38]. Further iterations of the measure may seek to consider whether increased inclusion of questions around protective factors may support use of the measure in clinical settings and understanding of recovery over time."

Reviewer 3	
1. In the introduction, it is not until around page 5-6 that we get some sense of the authors' definition of "older adults" and why they think recovery parameters may be different for this group (mention of beyond working age). It would help the siting of the research to introduce this much earlier.	Definition added to 2nd paragraph (page 5)<br><br>"Older adults have been defined as people aged 60+, a population who are more likely to experience reduced functioning, increased levels of cognitive impairment and higher levels of medical comorbidity (Chet et al., 2017)."
2. Throughout the manuscript, the language used to describe the participants who brought lived experience of bipolar disorder varies, with inconsistent descriptions of who took part where. In the methods, 4 participants are described as "knowledge users" and a distinct group from the "advisory group", but the latter is then described as including "experts by experience" with no elaboration on what that means. Knowledge users and experts by experience appear to be used interchangeably across all elements of the manuscript without clear definition of either, with the term "service user" also sometimes appearing. Further, the discussion section shifts to describing knowledge users as involved in all stages, which is not supported by the flow diagram. Suggest revising all parts to clearly define and consistent apply terms relating to lived experience.	Definition of knowledge users highlighted (page 6).<br><br>Removal of the term "experts by experience" across the paper to improve clarity.<br><br>Knowledge user label includes service users and academics. Change of names to service user group and research group across the paper to make this clearer.
3. The methods are fairly well described, with the exception of the analysis section. Content analysis is part of a broad family of methods that can be implemented in a number of ways - a reference to a specific process would help. The analysis needs to be further unpacked with details such as who conducted the analyses, did they involve frequencies, were they checked with	Please see additional information including that frequencies were recorded and who by. Re-wording of sentence indicating that analysis was checked by the service user group in the second focus group as this was not clear (page 9).

members to assist with interpretation etc.	
4. The main text of the results section is quite light on content and relies on cross-referencing to tables for an understanding of the key findings. This section would benefit from further incorporation of findings and perhaps some of the quotes within the narrative to give a deeper sense of the content of themes and feedback.	Quotes and further detail on findings included throughout results section (pages 11 to 12).
5. A key limitation of the study is the disproportionate lived experience versus clinical and academic involvement. The study is positioned as using IKT to integrate a range of views, but the authors do not explicitly address the unbalanced numbers and power dynamics apparent in 4 (+2?) people with bipolar disorder versus 15 advisory group members + 4 researchers from clinical and academic backgrounds. As the methods further describe the researchers conducting analyses and making decisions regarding contradictory feedback, the strength of the lived experience contributions as "collaboration" is questionable as they appear to have been filtered through clinical/academic lenses at each stage. This needs to be acknowledged throughout the manuscript and particularly as a limitation of the study.	Additional discussion in limitations section as well as comments made around this throughout the paper (Pages 10, 12, 15).
6. Page 9, please revise the phrasing "...explore the bipolar experiences..." to, for example, "explore the experiences of bipolar disorder".	Completed thank you (page 9).
7. Page 3 Limitations please revise "expert by experiences" to "experts by experience".	Phrase removed in line with previous revision.
8. Although subject of a separate paper under development, the comment on p13 regarding establishing validity needs some unpacking. The argument for the current work is that no suitable measure of recovery for older adults with bipolar disorder exists, so how will cross-	Additional comments added to this section (page 13).<br><br>"An IKT approach allows for development of a tool which aligns with the views of key stakeholders. Further comparison of the measure with validated tools which capture

validation and reliability be undertaken as separate to the face validity of the co-developed measure? Requiring traditional psychometrics appears to undermine the value of the IKT process.	concepts related to recovery can then indicate face and content validity of the questionnaire in capturing personal recovery experiences. Responses on the BRQ-OA will be compared to measures of mental health and wellbeing commonly associated with recovery in the literature. This can then support the paper in being both relevant to service user experiences and accurate in capturing concepts relevant to recovery."
